# Liraglutide: another reason to target prediabetes?

**DOI:** 10.18632/oncotarget.22256

**Published:** 2017-11-01

**Authors:** Conor F. Murphy, Neil G. Docherty, Carel W. le Roux

**Affiliations:** Carel W. le Roux: Diabetes Complications Research Centre, Conway Institute, University College Dublin, Ireland and Gastrosurgical Laboratory, Sahlgrenska Academy, University of Gothenburg, Gothenburg, Sweden

**Keywords:** prediabetes, liraglutide, obesity, SCALE, GLP-1

Glucagon-like peptide-1 (GLP-1) analogues, such as liraglutide, reduce bodyweight regardless of glycaemic status [1, 2], and are effective treatments for type 2 diabetes [3]. The anti-diabetic mechanism of action is based on enhancement of glucose-dependent insulin secretion, and an additional insulin sensitising effect characterised by weight loss and sustained reductions in HOMA-IR, and fasting insulin levels. There is evidence to suggest that lifestyle interventions with or without pharmacotherapy may reduce the risk of progression to diabetes in prediabetic individuals [4, 5]. Whether liraglutide is effective in this setting was the subject of a multi-national, double-blind, randomised controlled SCALE obesity and prediabetes trial [6]. This study targeted overweight, or obese, individuals with prediabetes. The primary outcome was the time to onset of diabetes over 160 weeks. A total of 2254 patients were randomised in a 2:1 format, to liraglutide 3.0mg, or placebo, both in conjunction with standardised diet and exercise. This study showed meaningful and sustained improvement in glycaemic control with reduced insulin resistance in the context of 6.1% weight loss over 3 years.

Only 3% in the Liraglutide treated group developed diabetes, compared to 11% in the placebo group, when the cumulative probability of a diagnosis was considered. Time from randomisation to diagnosis of diabetes in the treatment group was 2.7 times longer, equating to a hazard ratio of 0.21 (95% CI 0.13-0.34). In addition, at 160 weeks, 66% of patients in the liraglutide group had achieved normoglycaemia, compared to 36% with placebo [odds ratio of 3.6 (95% CI 3.0-4.4)].

Primary analyses were performed using last observation carried forward imputations, but as only 53% and 45% of the liraglutide and placebo group respectively completed 160 weeks follow-up this remains a limitation. This rate of attrition is consistent with previous weight loss studies, but mandates cautious data interpretation [7]. Rigorous sensitivity analyses aimed to optimise robustness of the data. An additional post-hoc analysis, at week 172, attempted to further address this issue of missing data, assuming undiagnosed diabetes in 1% of those withdrawn in the liraglutide group and 0% in the placebo group, and showed a 66% risk reduction [adjusted hazard ratio 0.34 (95% CI 0.22-0.53)].

There were more adverse events in the treatment group and a higher proportion of withdrawals due to adverse events (13% vs. 6%). Mild gastrointestinal side effects were a well-documented side-effect, but the recording of gallbladder associated events was a new finding. However, this effect did coincide with significant body weight loss, itself a known risk factor for gallstones.

The SCALE study design required a trade-off between inferring causation i.e. treatment efficacy, and the generalisability of the results. We can however deduce that daily liraglutide 3.0mg, in a population of patients who were overweight or obese and prediabetic, when taken for 160 weeks as an adjunct to diet and exercise, may facilitate sustained weight loss and reduce the risk of progression to diabetes over that time. Future studies will help to elucidate the significance of these effects with regards to patient-important outcomes, and to stratify the patients who will derive most benefit from therapies, facilitating targeted approaches, while reducing the number of patients who are exposed to side effects.

Consistent with this novel paradigm, the STRIVE study (NCT03036800) is a ‘real-world’ open-label RCT looking at the effect of adding liraglutide to standard of care as per a targeted prescribing pathway with efficacy targets, and prespecified stopping time-points should these not be met. STRIVE, following what has been learned through the SCALE program, will now assess the benefit of targeted treatment with liraglutide 3.0mg on effectiveness, budget impact and cost-effectiveness.

In conclusion, combining results from this and future studies will allow us to definitively establish how best to ensure judicial use of a potential anchor drug in the treatment of obesity and prediabetes.

## References

[1] Pi-Sunyer X (2015). N Engl J Med.

[2] Potts JE (2015). PLoS One.

[3] Marso SP (2016). N Engl J Med.

[4] Tuomilehto J (2001). N Engl J Med.

[5] Knowler WC (2002). N Engl J Med.

[6] le Roux CW (2017). Lancet.

[7] Fabricatore AN (2009). Obes Rev.

